# Alpha Reactivity to Complex Sounds Differs during REM Sleep and Wakefulness

**DOI:** 10.1371/journal.pone.0079989

**Published:** 2013-11-18

**Authors:** Perrine Ruby, Camille Blochet, Jean-Baptiste Eichenlaub, Olivier Bertrand, Dominique Morlet, Aurélie Bidet-Caulet

**Affiliations:** 1 Brain Dynamics and Cognition Team, Lyon Neuroscience Research Center (CRNL) - INSERM - CNRS, Lyon, France; 2 University Lyon 1, Lyon, France; University of Surrey, United Kingdom

## Abstract

We aimed at better understanding the brain mechanisms involved in the processing of alerting meaningful sounds during sleep, investigating alpha activity. During EEG acquisition, subjects were presented with a passive auditory oddball paradigm including rare complex sounds called Novels (the own first name - OWN, and an unfamiliar first name - OTHER) while they were watching a silent movie in the evening or sleeping at night. During the experimental night, the subjects’ quality of sleep was generally preserved. During wakefulness, the decrease in alpha power (8–12 Hz) induced by Novels was significantly larger for OWN than for OTHER at parietal electrodes, between 600 and 900 ms after stimulus onset. Conversely, during REM sleep, Novels induced an increase in alpha power (from 0 to 1200 ms at all electrodes), significantly larger for OWN than for OTHER at several parietal electrodes between 700 and 1200 ms after stimulus onset. These results show that complex sounds have a different effect on the alpha power during wakefulness (decrease) and during REM sleep (increase) and that OWN induce a specific effect in these two states. The increased alpha power induced by Novels during REM sleep may 1) correspond to a short and transient increase in arousal; in this case, our study provides an objective measure of the greater arousing power of OWN over OTHER, 2) indicate a cortical inhibition associated with sleep protection. These results suggest that alpha modulation could participate in the selection of stimuli to be further processed during sleep.

## Introduction

Sleep and wakefulness are two physiological states accompanied by different levels of consciousness. At sleep onset, the subject experiences a loss of consciousness. During sleep, reactivity to external stimulation is reduced and modified [1]–[3], and an oneiric consciousness (dreaming) with or without consciousness of the external world (external stimuli incorporation into dream) may arise [4], [5]. However, the brain mechanisms underlying these major phenomenological changes from wakefulness to sleep are not yet fully understood.

Several studies searched for the maintenance, during sleep, of electrophysiological markers associated with various perceptive/cognitive processes during wakefulness in human. So far, components like the N1 elicited by pure tones (marker of low-level auditory processing) [6], the MMN elicited by deviant pure tones (marker of sensory memory) [3], the P300 elicited by the own first name presented among other first names (marker of word discrimination) [7], the P3a, elicited by deviant pure tones (marker of attention orienting) [3], the N400 elicited by semantically incongruent pairs of words (marker of semantic processing) [8], were found to be maintained but modified during sleep. These studies strongly argued in favor of the maintenance of not only perceptive but also complex cognitive processes during sleep. Some behavioral results even suggest that the sleeping brain is able to select, according to its meaning, the information to be processed to potentially trigger awakening. Indeed, Adrian (1937) cited by Brain (1958) [9] and Formby (1967) [10] showed that a young mother is more easily awaken by her infant’s screams than by other sounds or other baby cries. These behavioral and electrophysiological data suggest that the sleeping brain can filter auditory information so that only alerting and/or significant stimuli reach higher-level processing.

We aimed at better understanding the brain mechanisms involved in higher-level processing of alerting meaningful sounds during sleep. During wakefulness, processing of alerting meaningful sounds is commonly studied in electroencephalography (EEG) using an Oddball Novelty paradigm. In this kind of protocol, subjects are presented with an auditory stream composed of frequent pure tones (Standards), random and less frequent slightly different pure tones (Deviants) and random, rare and very different sounds (called novel stimuli or “Novels”). In such a context, novel stimuli have a great alerting power due to their saliency and to their low frequency of presentation. To improve our understanding of the brain mechanisms involved in the selective processing of alerting and meaningful sounds during sleep, we thus investigated the brain activity induced by novel sounds. We chose to investigate alpha activity as a marker of brain activity for the following reasons.

In the sleep literature, the presence of alpha rhythm in the EEG is one of the criteria signaling the wakefulness state [11], [12]. More precisely, according to the criteria published by the American Sleep Disorders Association (ASDA) [13], an EEG arousal is an abrupt shift in EEG frequency during sleep, longer than 3 seconds, which may include theta, alpha and/or frequencies greater than 16 Hz but not spindles.

In the wakefulness literature, ongoing alpha rhythms were first discovered by Berger (1929) [14] when the subject is awake, with eyes closed. This result raised two hypotheses: (1) alpha would operate as an idling rhythm when alertness decreases, or (2) alpha would be related to processing of visual information. Moosmann et al. [15] showed later that ongoing alpha rhythms decrease at eyes opening even in the dark. This result supports the first hypothesis i.e. that alpha decrease would rather be induced by increased alertness than by stimulus appearance. Later studies then suggested that alpha decrease would be related to the processing of stimuli [16].

In the visual modality, decreases in the power of alpha rhythms (also called event-related desynchronization) were observed over regions involved in the task realized by the subject. By contrast increases were found over regions irrelevant to the task [17]. These results led authors to suggest that alpha rhythms would be involved in the active inhibition of the brain regions not involved in the current brain operations [17], [18]. Thus, a decrease in alpha power in a specific brain region would correspond to a release of inhibition and an increased excitability, enabling active processing [19]. In the auditory modality, few EEG studies investigated alpha oscillations. Taken together, they bring evidence that sounds induce an alpha decrease with a parietal topography [20]–[22], whereas the topography of alpha decrease induced by visual stimuli is more occipital [20]. Moreover, the more complex or task-relevant the stimuli, the greater and longer the alpha decreases, reflecting increased stimulus processing [20]–[24]. One study specifically investigated alpha rhythms, in response to the own first name during wakefulness [25]. In their paradigm, three stimuli were presented with equal probabilities, the subject’s own first name (Own), another first name (Other) and Own uttered backward (back-Own). Only Own stimuli induced a power decrease in the alpha band (8–13 Hz) at frontal electrodes, from 500 to 1000 ms after stimulus onset. Höller’s results suggest that the most significant stimulus reached higher processing level than the less significant ones [25]. As a whole, results in the auditory modality are in agreement with those obtained in the visual modality i.e. in both modalities a stimulus presentation induces a decrease in alpha power.

Therefore, during wakefulness, alpha rhythms seem to play an important inhibitory role involved in the selection of stimuli to be processed and brain regions to be activated (or not inhibited) [16]–[18]. Particularly, the alpha decrease seems to be related to the strength of stimulus processing.

Thus, in light of the functional role attributed to the alpha band modulation during sleep and wakefulness, we hypothesize that alpha could play an important role in the selection of external stimuli to be processed during sleep. However, few studies investigated spontaneous alpha oscillations during sleep [26]–[28], and to our knowledge no study investigated alpha rhythms induced by stimulations during sleep.

In the present study, our purpose was to investigate alpha oscillatory activity in response to complex sounds presented as Novels during sleep and wakefulness. Namely we aimed at identifying alpha modulations in response to significant Novels (the own first name – OWN) versus less significant Novels (an unfamiliar first name - OTHER) during wakefulness and sleep. To investigate oscillatory power in the alpha frequency-band we performed time-frequency analysis on EEG data previously used for ERPs analysis [29], [30]. We hypothesized that, during wakefulness, the two Novels would induce different levels of alpha decrease i.e. that the significant OWN would induce a larger decrease (indexing higher level processing) than the less significant OTHER. During sleep, we also expected the two Novels to induce different levels of alpha activity.

## Materials and Methods

### Subjects and Ethics Statement

Thirty-six healthy right-handed volunteers (18 males, mean age 23 years ±3), good sleepers, without hearing deficit and without medical, neurological, or psychiatric history participated in the study. In order to reduce stimuli disparities between subjects, one further inclusion criterion was a short first name: a majority of the subjects had a di-syllabic first name (27 subjects) and few of them had a first name with 3 or 4 syllables (9 subjects). Subjects were asked not to nap or consume energy drinks/food the day of the experiment.

The study was approved by the local ethical committee (Comité de protection des personnes SUD-EST II, hôpital Hotel-Dieu, Lyon, France), and subjects gave written informed consent according to the Declaration of Helsinki. Subjects were paid for their participation.

### Stimuli

The auditory stimuli were spectrally rich tones with a main frequency of 800 Hz and two harmonic partials (1600 and 3200 Hz) with different durations (Standards and Deviants), the subject’s own first name (OWN) and an unfamiliar first name (OTHER). For each subject, the choice of OTHER met strict criteria. Firstly, OTHER was chosen to sound equally unfamiliar for all subjects i.e. it was not the first name of a person in their close environment. This criterion was controlled thanks to the following procedure a) a questionnaire asking the subject to list the first names of the persons in his/her close environment (the questionnaire was filled in a couple of weeks before the recording session), b) a questionnaire presenting OTHER among several first names and asking the subject to indicate which first names belonged to a person in his/her close environment (this questionnaire was filled in few days before the recording session). Moreover, first names belonging to famous people were avoided. Secondly, we chose OTHER so that it could be perceived as different from OWN from the beginning of its utterance, while showing matching acoustic properties, more particularly at its onset. In order to obtain such a result, OTHER was chosen with the same first phoneme as OWN, the first syllables of the two names being however different. Thus, 1) OWN and OTHER differed from their onsets, since their first phonemes were uttered differently through the coarticulation phenomenon [31], 2) they showed a similar energy in their onset, illustrated by the fact that the root mean square (RMS) values of the two stimuli in the first 50 ms were found not different (3321±2074 for OWN, 3259±2235 for OTHER, paired t-test: t(35) = 0.45, p>0.05). Additionally, OTHER was chosen to present the same number of syllables as OWN (e.g. OWN: Thomas, OTHER: Tanguy). Altogether, this operating mode allowed us to obtain an OTHER stimulus that is perceived as different from OWN from its onset, while minimizing the dissimilarities between the envelops of the acoustic signal plots of the two stimuli.

First names were digitally recorded by a neutral masculine voice using Adobe Audition 1.5 (Adobe software). After recording, maximum amplitudes of all stimuli were normalized. The mean durations of OWN (581 ms ±86) and OTHER (598 ms ±78) were not significantly different [29], [30].

### Experimental Design

The presentation of the four types of auditory stimuli obeyed the rules of a novelty oddball paradigm. Spectrally rich tones lasting 75 ms and 30 ms (including 5 ms rise/fall times) were used respectively as Standards (p = 0.82) and Deviants (p = 0.14). OWN and OTHER were presented as novel stimuli and the probability of occurrence was 0.02 for each of them. The stimuli were presented in a pseudo-randomized order so that 1) each Deviant followed at least two Standards, 2) each Novel followed at least ten tones, and 3) Novels were always preceded by a standard tone. Stimulus onset asynchrony (SOA) was set at 650 ms, except for the Standard following a Novel, which appeared 1260 ms after the Novel onset, whatever the duration of the Novel [29], [30].

### Procedure

Subjects arrived in the lab at 7.00 p.m. after they had eaten. During approximately one hour and a half, electrodes were fixed on their head and face. The subjects selected a movie among a choice of comedy or action movies. Then, subjects were installed in an acoustically dampened and electrically shielded room, earphones were inserted in their ears, and their hearing threshold was assessed using standard stimuli. The evening recording session started at 10.24 p.m. ±45 min (duration, 1 hour and 6 min ±9 min). Stimuli (around 120 Novels were presented in average) were presented binaurally at 50 dB above the subject’s hearing level using the Presentation software (Neurobehavioral Systems). Subjects were instructed to watch the movie (silenced with subtitles) and to ignore the auditory stimuli [29], [30]. During the night session, stimuli were continuously presented (around 930 Novels were presented in average), as subjects were in bed [29].

### Electrophysiological Recordings

Twenty-one Ag/AgCl scalp electrodes were manually positioned according to the extended International 10–20 System (Fz, Cz, Pz; FP1, F3, FC1, C3, T3, CP1, P3, M1, O1 and their counterparts on the right hemiscalp). This relatively small number of electrodes was both compatible with sleep recordings and with the use of scalp potential maps. We concentrated the electrodes around the sites expected to show between first name effects i.e. central and parietal sites. Contact between skin and electrodes was made using EC2 electrode cream Pactronic (Grass Product Group) and electrodes were fixed on the scalp using the paste TENSIVE (Parker Laboratories, Inc.). The reference electrode was placed on the tip of the nose, and the ground electrode on the forehead. The electro-oculogram (EOG) was recorded from 2 electrodes placed on the supraorbital ridge of the left eye and on the infraorbital ridge of the right eye. Muscle activity (EMG) was recorded from 2 electrodes attached to the chin. Electrode impedance was kept below 5 kΩ. The electrophysiological data (EEG, EOG and EMG) were continuously recorded via a BrainAmp system (Brain Products GmbH, Germany) with an amplification gain of 12 500, a high-pass filter of 0.1 Hz with a 12 dB/octave slope, and a sampling rate of 1000 Hz with an anti-aliasing low-pass filter (cutoff frequency being 250 Hz with a 3 dB/octave slope).

### Sleep Stage Scoring

Sleep stages were scored off-line, visually by JBE according to standard criteria [12], and automatically by ASEEGA software (http://aseegaonline.com/pub/index.html) [32] to derive hypnograms based on 30 s epochs and to determine the vigilance state (wake, rapid eyes movements sleep - REM sleep, sleep stage 1 - N1, sleep stage 2 - N2 and slow wave sleep - N3) that occurred for every stimulus delivered during sleep. Only the sleep periods for which JBE and ASEEGA scores agreed were considered for analysis. The percentage of agreement between JBE and ASEEGA respective scoring was 82.9% with a kappa coefficient of 0.762 (epoch-by-epoch comparison; epochs scored as artefacts were excluded from the statistical analysis).

### Time-Frequency Analysis

Analysis was focused on responses to OWN and OTHER stimuli and on the alpha frequency band. In the literature the frequency range of the alpha band is classically 8–12 Hz (e.g. [33], [34]), but some authors consider wider bands between 7 and 13.5 Hz [17]. Therefore, we used two approaches: (1) analyses within the 8–12 Hz frequency band which are presented in the main article, and (2) analyses within a larger 7–12 Hz frequency range, based on the individual alpha frequencies found during wakefulness and REM-sleep, which are presented in File S1.

Oscillatory activities induced by the two novel stimuli were characterized separately in each vigilance stage. Before analysis, trials were automatically excluded from analysis when the overall electrophysiological signal amplitude exceeded 150 µV during wakefulness, 125 µV in REM sleep, and 400 µV in N2 and N3.

We investigated oscillatory activities by means of wavelet decomposition, which provides a good compromise between time and frequency resolutions. We used complex Gaussian Morlet’s wavelets (complex waves with a Gaussian shape in the time- and frequency- domains) with a ratio f/σ_f_ = 7, f being the central frequency of the wavelet and σ_f_ the standard deviation of the Gaussian envelop in the frequency domain [35]. With these parameters, the 10 Hz wavelet shows a bandwidth of 2.9 Hz in the frequency domain and a duration of 220 ms in the time domain. Each single trial signal was transformed in the time-frequency (TF) domain by convolution with the complex Morlet’s wavelets with a 1 Hz frequency step on the time-window from −1000 to 2000 ms around stimulus onset. Averaging these TF powers would result in a power estimate of both evoked (phase-locked to stimulus onset) and induced (jittering in latency) activities in the TF domain, which reflect different neural mechanisms [35]–[37]. To reduce the contribution of stimulus phase-locked responses, for each subject, we averaged evoked potentials on the same time-window, and we subtracted this average from each single trial before time-frequency transformation. Then, the time-frequency responses of such corrected single trials were grand averaged across trials for each subject to get an estimate of the induced oscillation in the TF domain with few or no contamination by evoked activities.

We analyzed the oscillation power on the {−500; 1500 ms} time-window around each stimulus onset. For visualization purposes only, a baseline correction was applied to all stimuli by subtracting the mean power between −300 and −100 ms before stimuli onset, in each frequency band.

### Statistical Analysis

As we were interested in modulations of alpha oscillations, we performed statistical analysis across subjects on the averaged power in the 8–12 Hz frequency band. Moreover, as the Standard after a novel stimulus occurred 1260 ms after its onset we performed statistics on 200-ms time-windows between 0 to 1200 ms after novel onset.

To limit assumption on the data distribution, we used a statistical permutation test based on randomizations [38]. Each randomization consisted in (1) the random permutation of the 36 pairs (corresponding to the 36 subjects) of values, (2) the sum of squared sums of values in each of the 2 obtained samples, and (3) the computation of the difference between these two statistic values. We did 100 000 such randomizations to obtain an estimate of the distribution of this difference under the null hypothesis. We then compared the actual difference between the values in the 2 conditions of interest to this distribution.

Alpha reactivity to each Novel (OWN or OTHER) was detected using permutation tests comparing the mean power in the 8–12 Hz frequency band over 200 ms windows moving by step of 100 ms (from 0 to 1200 ms) with the pre-stimulus power (mean power over a window between −300 and −100 ms before stimulus onset). These time-parameters are justified by the 220 ms duration of the 10 Hz wavelet. To identify with no prior assumption the location and latency of the effects, we performed randomization tests for each of the 21 electrodes on successive 200 ms time-windows, and corrected for multiples tests. In the temporal dimension, we used a randomization procedure [39] to estimate the minimum number of consecutive time-windows that must be significant to detect an effect in the entire time-period of interest. In the spatial dimension, we used a cluster-based approach and considered as significant an effect visible at 2 or more adjacent electrodes (e.g. [40], [41]).

To compare alpha power induced by OWN and OTHER stimuli, permutation tests were performed at the electrodes and time-windows showing a significant alpha reactivity to one of the Novels at least. We compared alpha power values between novel types without applying any baseline correction.

All analyses were performed with the ELAN Pack software developed at the Brain Dynamics and Cognition Team of the Lyon Neuroscience Research Center, Lyon, France (http://elan.lyon.inserm.fr) [42].

## Results

### Behavioural Results

Despite an uncomfortable experimental setup, the subject’s sleep quality was generally preserved, as observed from a comparison with the standard values of sleep parameters [43] (Table 1).

**Table 1:** Characteristics of the subject’s sleep during the experimental night measured by one scorer (JBE).

Sleep parameters	Experimental night values	Standard values
**Time in bed**: TIB (min)	464±57	390–510
**Sleep Period Time**: SPT (min)	439±49	
**Wakefulness during total sleep period**: W (min)	22±21	20–30
**Total sleep time**: TST = SPT−W (min)	417±54	
**N2 onset latency** (min)	24±21	20–30
**N1** **duration** (% of TST)	3±3	5–10
**N2** **duration** (% of TST)	40±8	40–55
**N3** **duration** (% of TST)	36±8	25–30
**REM sleep** **duration** (% of TST)	21±5	20–25

**Time in bed (TIB)**, time from lights off to lights on; **Sleep period time (SPT),** time from the beginning of the first episode of sleep to the end of the last episode of sleep; **Wakefulness during SPT**, amount of wakefulness during SPT; **Total sleep time (TST)**, SPT minus Wakefulness during SPT. Mean ± Standard deviation of the experimental values are presented. The range of standard values is given from Hirshkowitz et al. 2004. min: minutes.

### Time-frequency Analysis

The average number of accepted trials was 78±19 and 67±20 for OWN and OTHER respectively during wakefulness, 199±59 and 198±63 during sleep stage N2, 134±35 and 134±34 during sleep stage N3 and 120±36 and 120±35 during REM sleep.

### Reactivity of Alpha Rhythm (8–12 Hz) to OWN and OTHER

#### Wakefulness

**Figure 1:**
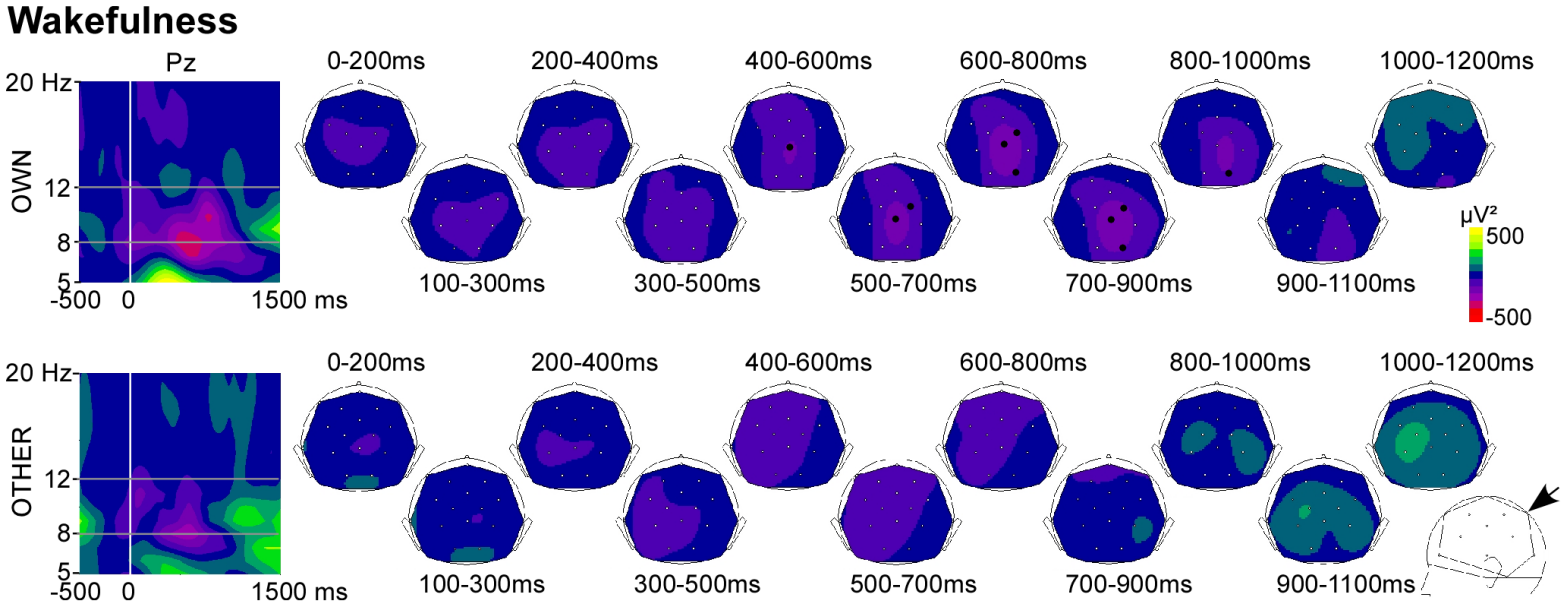
Alpha activity induced by novel stimuli during wakefulness. *Left panel*. Time-frequency analysis (TF) plots for OWN and OTHER at the electrode Pz, after baseline correction (baseline defined from −300 to −100 ms). x-axis : time, y-axis : frequency, the vertical white line indicates stimulus onset and the gray lines indicate the 8–12 Hz frequency band. *Right panel*. Scalp topographies (back-top views) of alpha power are presented in each tested 200 ms time window. The color scale represents in red (negative values) a decrease in oscillatory power compared to the baseline level, and in yellow (positive values) an increase. Black dots on the topographies indicate electrodes showing a significant alpha increase or decrease compared to baseline. In the bottom right corner, the arrow indicates the view angle used to generate the back-top topographies.

During wakefulness, a significant decrease in alpha power was observed in response to OWN at parieto-occipital sites (Pz: 400–900 ms; Cp2∶500–900 ms; O2∶600–1000 ms; p<0.05). OTHER did not induce any significant decrease in alpha power (Figure 1).

#### Non REM sleep (NREM)

**Figure 2:**
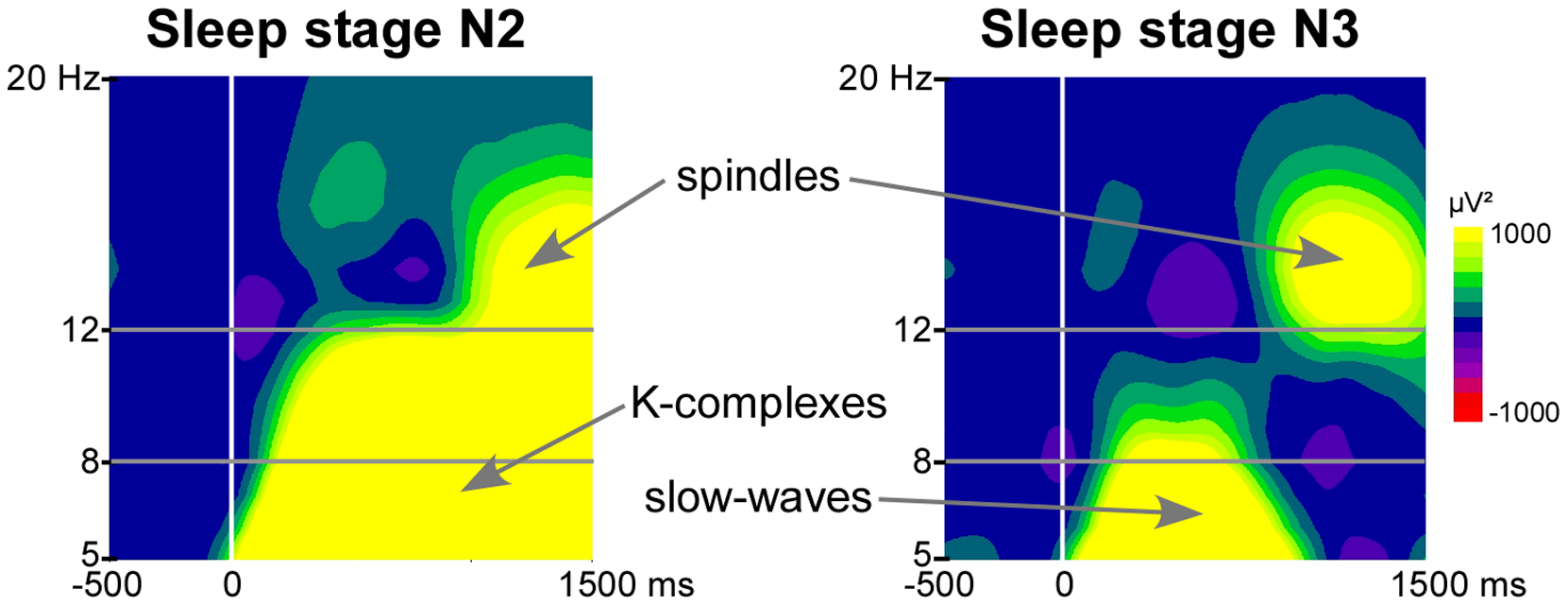
Alpha activity induced by OWN during sleep stages N2 and N3. Time-frequency analysis (TF) plots for OWN at electrode Pz, after baseline correction (baseline defined from −300 to −100 ms). x-axis: time, y-axis: frequency, the vertical white line indicates stimulus onset and the horizontal gray lines delineate the alpha frequency band (8–12 Hz).

During sleep stage N2 and sleep stage N3, a large power increase in response to each Novel was found significant at all electrodes, with a maximum over parietal electrodes (see an example for electrode Pz in Figure 2). During sleep stage N2, this increase was visually observable in the average over all subjects on two large frequency-bands (from 5 to 12 Hz and from 15 to 20 Hz) and on a large time-window from 200 to 1200 ms after Novel onset as described in previous studies [44]. During sleep stage N3, the power increase was observed between 5 and 11 Hz from 100 to 1000 ms and between 10 and 20 Hz from 800 to 1200 ms after Novel onset. These strong power increases most likely correspond to the frequency content of typical activities of NREM sleep, namely K complexes, spindles and slow waves [44]. We unsuccessfully tried to remove these typical sleep-related waves from the analyzed trials. Lowering the rejection threshold during the artifact rejection phase led to retain a number of clean trials too small for a safe analysis. As a consequence, due to the strong overlap of the spectrum of K complexes, spindles and slow waves with the alpha band (8–12 Hz), the genuine alpha reactivity to OWN and OTHER stimuli could not be correctly identified.

#### REM sleep

**Figure 3:**
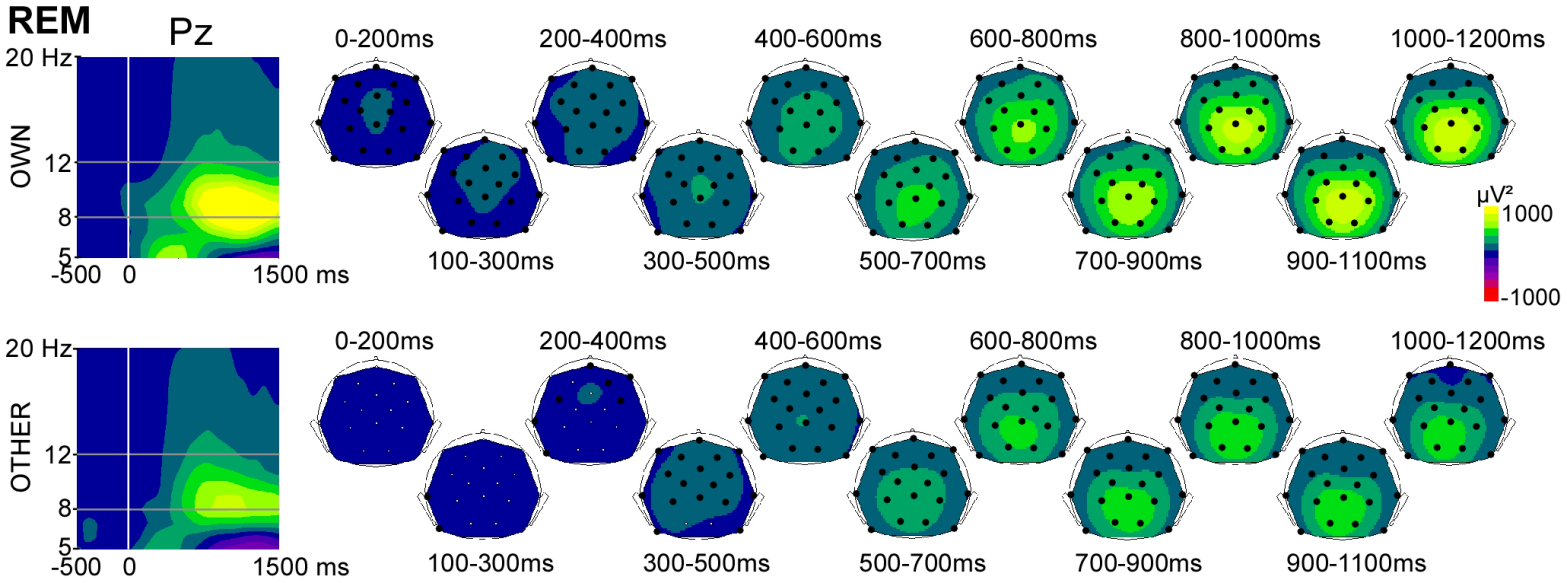
Alpha activity induced by novel stimuli during REM sleep. *Left panel*. Time-frequency analysis (TF) plots for OWN and OTHER at the electrode Pz, after baseline correction (baseline defined from −300 to −100 ms). x-axis : time, y-axis : frequency, the vertical white line indicates stimulus onset and the gray lines indicate the 8–12 Hz frequency band. *Right panel*. Scalp topographies (back-top views) of alpha power are presented in each tested 200 ms time window. The color scale represents in red (negative values) a decrease in oscillatory power compared to the baseline level, and in yellow (positive values) an increase. Black dots on the topographies indicate electrodes showing a significant alpha increase or decrease compared to baseline.

During REM sleep, a large increase in alpha power was detected for both Novels at all electrodes, with a maximum over parietal electrodes (Figure 3). The increase of alpha power started at stimulus onset in response to OWN and at 100 ms post stimulus in response to OTHER (p<0.01).

### Comparison of Alpha Activity (8–12 Hz) Induced by OWN and by OTHER

#### Wakefulness

**Figure 4:**
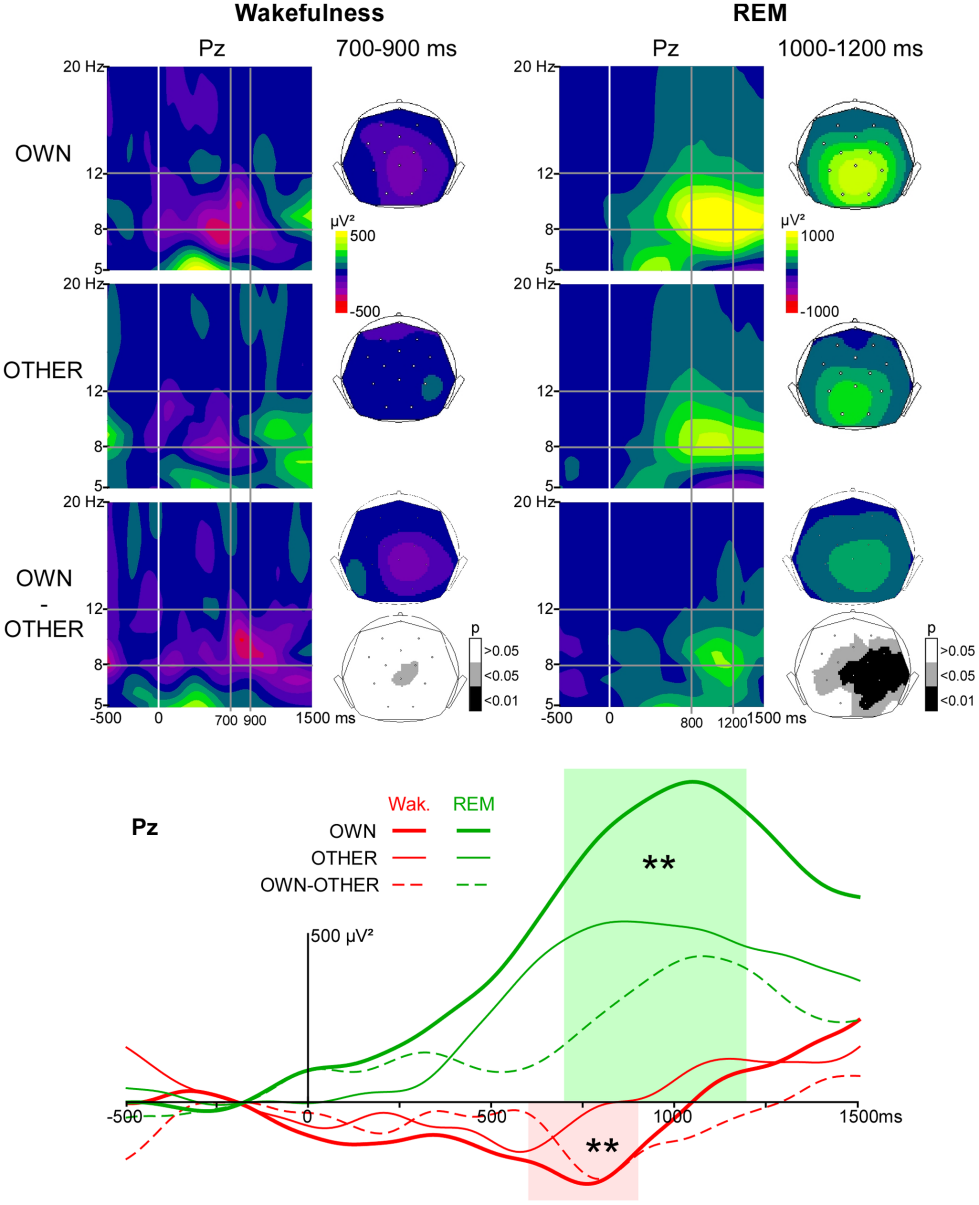
Alpha activity induced by novel stimuli. *Upper panel*. Time-Frequency (TF) plots for OWN, OTHER and OWN minus OTHER in the wakefulness and REM sleep conditions at the electrode Pz, after baseline correction (baseline defined from −300 to −100 ms). x-axis: time, y-axis: frequency, the vertical white line indicates stimulus onset and the horizontal gray lines indicate the time windows showing a significant difference between OWN and OTHER in the 8–12 Hz frequency band. Scalp topographies (back-top views) of alpha power are presented in the 700–900 ms time window during wakefulness and in the 1000–1200 ms time window during REM sleep. The color scale represents in red (negative values) a decrease in oscillatory power compared to the baseline level, and in yellow (positives values) an increase. The scalp topographies with a gray scale indicate electrodes showing a significantly different alpha power for OWN vs OTHER (p<0.05). *Lower panel*. Time profiles of TF power in the 8–12 Hz frequency band at the electrode Pz for OWN (bold continuous line), OTHER (thin continuous line) and OWN minus OTHER (dotted line) in the wakefulness (red) and in the REM sleep condition (green). Colored areas indicate the time windows with a significant difference in alpha power between OWN and OTHER during wakefulness (in red) and REM sleep (in green). The next stimulus (a standard tone) is presented 1260 ms after the first names onsets.

The decrease in alpha power was found to be significantly larger for OWN than for OTHER (p<0.05), at electrodes Pz between 600 and 900 ms, and CP2 between 700 and 900 ms after stimulus onset (Figure 4).

#### REM sleep

The increase in alpha power was found to be significantly larger for OWN than for OTHER (p<0.05), from 700 to 1200 ms after stimulus onset at electrodes Pz, C4, P4, CP1, CP2; from 700 to 1000 ms at electrode C3; from 800 to 1200 ms at Cz; from 900 to 1200 ms at 02; and from 1000 to 1200 at P3 (Figure 4).

In both wakefulness and REM sleep, no significant difference was observed in the pre-stimulus period between OWN and OTHER conditions.

Similar results are obtained with the 7–12 Hz frequency band (File S1).

## Discussion

The present study aimed at better understanding the brain mechanisms involved in the selective processing of alerting significant sounds during sleep. We investigated alpha oscillatory activity (8–12 Hz) in response to the own first name presented as an auditory novel stimulus in a stream of simple tones during sleep and wakefulness. In 36 healthy subjects, we compared the brain responses to the significant own first name (OWN) and to one unfamiliar and thus less significant first name (OTHER). First names were presented at equal low frequencies (2% of stimuli) in a novelty oddball paradigm while subjects were watching a silent movie with subtitles in the evening and during sleep at night. Our objectives were to investigate the reactivity of alpha rhythms induced by novel stimuli during wakefulness and sleep, and to assess whether the significance of the stimulus could affect alpha rhythms during sleep.

### Reactivity of Alpha Rhythm to Alerting Significant Sounds

#### Wakefulness

During wakefulness, a decrease in alpha power was observed, at parieto-occipital electrodes, from 400 to 1000 ms after the onset of the own first name. This result is consistent with the few EEG studies that have investigated alpha oscillation reactivity to sounds. An alpha decrease between 500 ms and 1000 ms, maximal at parietal electrodes, has been found in response to target pure tones [22], and to words during a semantic task [21].

Interestingly, Mazaheri & Picton (2005) [20] showed that alpha decrease in response to visual or auditory targets present different scalp topographies: an occipital topography maximal at O1 and O2 electrodes in response to visual targets and a more widespread parietal topography in response to auditory targets. These results show that alpha decrease induced by relevant auditory stimuli present a parietal topography using scalp EEG.

In the present study, participants were hearing sounds while watching a movie. Thus, they were presented with both auditory and visual stimulation, and the alpha decrease could be related to either (1) auditory processing; or (2) visual processing associated with reorientation of attention toward the movie. The parietal rather than occipital topography and the timing of the alpha decrease (an alpha decrease related to a reorientation of attention toward the movie would be expected to be more delayed) argue in favor of the first hypothesis.

From the widespread parietal topography of the alpha decrease, it is difficult to make precise assumption on the underlying brain sources. Indeed, parietal topographies can result from activity just beneath the electrodes in the parietal cortex, but also from activity at distance in the superior temporal cortices (auditory cortices) according to the dipole orientation. Thus, the parietal topography of alpha decrease is compatible with sources (1) either in the auditory cortices, as observed in response to passively heard syllables using electrocorticography (ECoG) recordings [36], in response to words during an intelligibility task using EEG [45], in response to auditory targets using MEG [46], and as reviewed by Weisz et al. (2011) [24], (2) or in the parietal cortex. According to the inhibition-timing hypothesis [17], a decrease in alpha power in a specific brain region would reflect a release of inhibition and an increased excitability, facilitating active processing within this brain area [19]. Thus, the parietal alpha decrease could reflect an increased excitability either of auditory cortices, or of parietal brain areas. In the latter case, the alpha decrease in response to auditory Novels could index several high-level processes [17], such as selective attention [18], [47], semantic processing [21] of the Novels, or retrieval of episodic memories [48]–[50] associated with the Novels. Therefore, although we cannot specify the brain origin of the alpha decrease, we can still argue that its functional role is most likely related to an increased active processing (by a release of inhibition) in brain areas involved in the processing of the novel sounds.

#### Non REM sleep

By contrast with wakefulness, during NREM sleep, a power increase was found in the frequency band of interest (8–12 Hz). Such increase seems mainly due to the characteristic features of NREM sleep such as K-complexes, slow waves and spindles. K-complexes are known to be evoked or induced by auditory stimulation [51]–[53], and as slow waves, they are slow components with a broad spectrum including frequencies in the alpha band. As a consequence in the TF plots of sleep stage N2 and sleep stage N3, the increased power in the 5–12 Hz band is most probably due to stimulus-related K-complexes and slow waves, respectively, and the increased power in the 10–20 Hz band is most probably due to subsequent spindles (Figure 2).

Note that, little is known about stimulus-related slow waves during sleep stage N3 because only few studies reported ERP results in deep sleep [3], [54] and none of them used complex stimuli. Concerning spindles, Sato et al. (2007) [55] reported that during a period of sensory stimulation, spindle frequency and duration increased compared to a period without stimulation. However, to our knowledge, no study reported spindles induced or evoked by auditory stimulation. The increased amount of spindles around 1000 ms post stimuli in our study may be due to the fact that K-complexes are often followed by a spindle in natural sleep.

As a consequence, in our results if a change in genuine alpha rhythm was induced by the stimuli, we could not disentangle it from the characteristic sleep features.

#### REM sleep

During REM sleep, an important increase in alpha power was found in response to both Novels. By contrast with NREM sleep such an increase could not be explained by K-complexes, slow waves or spindles [11], and may be a genuine alpha increase (the peak of power increase is in the 8–10 Hz band). Interestingly, this new result shows that the alpha reactivity to stimuli has two opposite directions during wakefulness (decrease) and REM sleep (increase).

Cantero et al. (2002) [27] proposed that increased alpha power during REM sleep would reveal micro-arousal without awakening. They hypothesized that micro-arousals facilitate the momentary contact with the external environment without alterations in the REM continuity [27]. Some recent results argue in favor of this hypothesis. By systematically challenging sleep with realistic and varied acoustic disruption, McKinney et al. (2011) [56] found that sleepers exhibited markedly greater sensitivity to sounds during moments of elevated alpha expression during non REM sleep. According to Cantero’s hypothesis, the increase of alpha power induced by Novels in our study could reveal their disturbing impact and could reflect micro-arousals without awakening [27]. Yet, the functional role attributed to alpha rhythms in REM sleep by Cantero et al.’s hypothesis is not consistent with their functional role during wakefulness according to the inhibition-timing hypothesis [17], [18]. According to the wakefulness literature, an increase in alpha power would rather be associated with cortical inhibition than with cortical excitation. In agreement with this hypothesis, Benca et al. (1999) [26] suggested that increased alpha power might represent cortical deactivation during sleep. If we interpret our results in line with the latter hypothesis, the increased alpha power in response to Novels would be associated with an inhibition process resulting in a diminished processing of the Novels. This reduced processing may result in a protection of sleep, preventing the subject from awakening. This hypothesis is compatible with the high auditory awakening threshold observed during REM sleep (auditory awakening threshold is higher in REM sleep as compared to sleep stage N2) [57]. Note that in our experiment, when the subjects were allowed to sleep they had already heard the auditory paradigm for one hour. As a consequence, the Novels may have been perceived much more as disturbing rather than as alerting stimuli. It is likely that in such circumstances the brain develops an inhibiting strategy in order to preserve sleep. However, we can infer that if first names, and especially the own first name, would have been uttered once during silent REM sleep, brain processes triggered by this stimulus might have been different and could have led to subject’s awakening.

### Specific Alpha Activity Induced by the Own First Name

#### Wakefulness

During wakefulness, a greater alpha decrease was found in response to OWN compared to OTHER on parietal electrodes from 700 to 900 ms. So far, only few research groups specifically investigated induced alpha rhythms in response to the own first name during wakefulness [25], [58], [59]. They also found a larger power decrease for the own first name than for other first names [25], [59]. Our findings extend previous results as we found similar results in a different context of stimulus presentation. In previous studies the own first name and several other first names or complex stimuli were presented with equal probabilities [25], [59], whereas in our study OWN and OTHER were rarely presented, as Novels, within a stream of simple standard and deviant sounds, enhancing the alerting power of OWN and OTHER.

The larger alpha decrease for OWN compared to OTHER is consistent with the hypothesis that an alpha decrease in response to meaningful sounds would be related to an increased active processing of the stimuli (for reviews, see [16], [19]). Indeed, the own first name is more significant to the subject than an unfamiliar first name. In addition, studies in experimental psychology have shown that the own first name is more powerful than another word or first name to involuntarily capture attention [60], [61] and electrophysiological studies have shown that it could induce more higher-level processing than another unfamiliar first name [30]. Previous studies showed that alpha decrease in response to sounds is larger when the stimulus is task-relevant [20], [22] or when the task requires higher-level processes such as semantic processing and selective attention [16], [21], [23], [24]. Our result extends these findings by showing that the alpha decrease is larger in response to sounds with more significance and alerting power such as the own first name even during passive listening. This result is in agreement with the “information via desynchronization” hypothesis proposing that neural desynchronization is positively related to the richness of information to be processed [19]. Taken together, these results suggest that during wakefulness the amplitude of the alpha decrease is related to the significance of the incoming stimulus (either task relevance or intrinsic content).

#### REM sleep

During REM sleep a greater alpha power increase was induced by OWN than by OTHER from 700 to 1200 ms. According to Cantero’s hypothesis, this result would reveal that the own first name is more powerful than another first name to disturb sleep and to increase the arousal level of the subject. By contrast, if alpha rhythms are associated with inhibition during sleep, this result would mean that OWN was more inhibited than OTHER. This may be linked to the fact that OWN is more alerting/significant than OTHER. As a consequence a greater inhibition would be needed to reduce the processing of OWN. Indeed, according to Muzet (2006) [62] the sleeping subject’s reactivity toward an auditory stimulation would not be proportional to its intensity but rather to its significance.

Finally, since this is the first study with time-frequency analysis of oscillatory activities induced by complex sounds during sleep, further studies are needed to bring new insight on the functional meaning of alpha reactivity during sleep as well as on the mechanisms of the sleeping brain to protect sleep from external stimuli.

### Conclusions

Our study shows that complex sounds have a different effect on the alpha power during wakefulness (decrease) and during REM sleep (increase). The increased alpha power induced by Novels during REM sleep may 1) correspond to a short and transient increase in arousal [27]; 2) indicate a cortical inhibition [16], [17] associated with sleep protection.

In addition, our data show that, when presented rarely and unexpectedly (as Novels), the own first name modulates the power of alpha oscillations during wakefulness as well as during REM sleep. During wakefulness, only the own first name induces a decrease in alpha power which could reveal, according to the current hypotheses [16], [17], [19], further active processing of the own first name compared to the unfamiliar first name. During REM sleep, the own first name induces a larger increase in alpha power than an unfamiliar first name. This effect may indicate 1) an increase in arousal according to Cantero’s hypothesis, and in this case, our study provides an objective measure of the greater arousing power of the own first name over another first name, 2) an increase in inhibition of the brain areas involved in sound processing i.e. an improved sleep protection.

Our study, which is the first one to study oscillatory activity induced by sounds during sleep, suggests that alpha modulations participate in the selection of stimuli to be further processed during sleep.

## Supporting Information

File S1 Supporting information. Supporting information includes supplementary methods and supplementary results related to the analysis of the 7–12 Hz frequency band.
(DOC)
