# Supplementary material for: Alpha Reactivity to Complex Sounds Differs during REM Sleep and Wakefulness
Source: PLoS One. 2013 Nov 18;8(11):e79989. doi: 10.1371/journal.pone.0079989 (PMC3832371; doi:10.1371/journal.pone.0079989)
Supplement: File S1 — Supporting information. Supporting information includes supplementary methods and supplementary results related to the analysis of the 7–12 Hz frequency band. (DOC) [file pone.0079989.s001.doc]

**Supplement to**

**« Alpha Reactivity to Complex Sounds Differs during REM Sleep and Wakefulness »**

Perrine Ruby, Camille Blochet, Jean-Baptiste Eichenlaub, Olivier Bertrand, Dominique Morlet and Aurélie Bidet-Caulet

**Supplementary method**

In a second analysis, we used the individual alpha frequencies to set the alpha range at the group level. We identified the frequency of the alpha desynchronization in each subject (we considered only the response to the own first name - OWN). To do so, we computed the frequency spectrum from the time-frequency power to OWN in the 0-1200 ms window after baseline correction for each subject, and detected the frequency of the trough in the 6-14Hz frequency range during wakefulness. The trough was found in the 8-12Hz frequency range in 35/36 subjects and was at 7Hz for the remaining subject. We also identified in each subject the peak frequency of the alpha synchronization to the own first names during REM sleep. The frequency of the maximum alpha synchronization was found in the 7-10Hz frequency range in all subjects. The intra individual difference between the desynchronization peak frequency during wakefulness and the synchronization peak frequency during REM sleep was in average 0.8 Hz ± 1.7 (SDV). These analyses highlighted that the alpha range common to all subjects during wakefulness and REM sleep was 7-12Hz.

**Supplementary results**

**Reactivity of alpha rhythm (7-12Hz) to OWN and OTHER**

**Wakefulness**. During wakefulness, a significant decrease in alpha power was observed in response to OWN at parieto-occipital sites (Cz: 500-1000 ms; Pz: 400-900 ms; Cp2: 400-1000 ms; O1: 400-900 ms; O2: 200-1000 ms; p < 0.05). OTHER did not induce significant decrease in alpha power.

**REM sleep**. During REM sleep, a large increase in alpha power was detected for both Novels at all electrodes, with a maximum over parietal electrodes. The increase of alpha power started at stimulus onset in response to OWN and at 200 ms post stimulus in response to OTHER (p < 0.01).

**Comparison of alpha activity (7-12Hz) induced by OWN and by OTHER**

**Wakefulness.** The decrease in alpha power was found to be significantly larger for OWN than for OTHER (p < 0.05), at electrodes Pz and CP2 between 700 and 900 ms after stimulus onset.

**REM sleep**. The increase in alpha power was found to be significantly larger for OWN than for OTHER (p<0.01), from 700 to 1200 ms after stimulus onset at electrode Pz, C4 and CP2 and from 800 to 1200 ms at electrode T4, O2 and P4.

In both wakefulness and REM sleep, no significant difference was observed in the pre-stimulus period between OWN and OTHER conditions.
